# Validation of markerless video-based gait analysis using pose estimation in toddlers with and without neurodevelopmental disorders

**DOI:** 10.3389/fdgth.2025.1542012

**Published:** 2025-02-25

**Authors:** Jeffrey T. Anderson, Jan Stenum, Ryan T. Roemmich, Rujuta B. Wilson

**Affiliations:** ^1^ Department of Medicine, University of California, Los Angeles, CA, United States; ^2^ Department of Physical Medicine and Rehabilitation, The Johns Hopkins University School of Medicine, Baltimore, MD, United States; ^3^ Center for Movement Studies, Kennedy Krieger Institute, Baltimore, MD, United States; ^4^ Semel Institute for Neuroscience and Human Behavior, Los Angeles, CA, United States

**Keywords:** neurodevelopmental disability, motor, pose estimation, validity, pediatric, gait analysis, markerless

## Abstract

**Introduction:**

The onset of locomotion is a critical motor milestone in early childhood and increases engagement with the environment. Toddlers with neurodevelopmental disabilities often have atypical motor development that impacts later outcomes. Video-based gait analysis using pose estimation offers an alternative to standardized motor assessments which are subjective and difficult to ascertain in some populations, yet very little work has been done to determine its accuracy in young children. To fill this gap, this study aims to assess the feasibility and accuracy of pose estimation for gait analysis in children with a range of developmental levels.

**Methods:**

We analyzed the overground gait of 112 toddlers (M: 30 months, SD: 8 months) with and without developmental disabilities using the ProtoKinetics Zeno Walkway system. Simultaneously recorded videos were processed in OpenPose to perform pose estimation and a custom MATLAB workflow to calculate average spatiotemporal gait parameters. Pearson correlations were used to compare OpenPose with the Zeno Walkway for velocity, step length, and step time. A Bland-Altman analysis (difference vs. average) was used to assess the agreement between methodologies and determine the difference of means. Developmental levels were assessed using the Mullen Scales of Early Learning.

**Results:**

Our analysis included children with autism (*n* = 77), non-autism developmental concerns (*n* = 6), tuberous sclerosis complex (*n* = 13), 22q deletion (*n* = 1), and typical development (*n* = 15). Mullen early learning composite scores ranged from 49 to 95 (m = 80.91, sd = 26.68). Velocity (r = 0.87, *p* < 0.0001), step length (r = 0.79, *p* < 0.0001), and step time (r = 0.96, *p* < 0.0001) were all highly correlated between OpenPose and the Zeno Walkway, with an absolute difference of means of 0.04 m/s, 0.03 m, and 0.01 s, respectively.

**Discussion:**

Our results suggest that video-based gait analysis using pose estimation is accurate in toddlers with a range of developmental levels. Video-based gait analysis is low cost and can be implemented for remote data collection in natural environments such as a participant's home. These advantages open possibilities for using repeated measures to increase our knowledge of how gait ability changes over time in pediatric populations and improve clinical screening tools, particularly in those with neurodevelopmental disabilities who exhibit motor impairments.

## Introduction

Motor development in early childhood is dynamic and allows a toddler to receive numerous learning opportunities through environmental exploration and social interactions. In toddlers with neurodevelopmental disorders (NDDs), aspects of atypical motor development such as delayed motor milestones, atypical gait, poor coordination, and balance have been widely described (1). These motor impairments are particularly prominent in children with genetic NDDs, often present earlier in life, and can be related to genetic severity (2, 3). Studies have also shown that motor impairments in those with and without neurodevelopmental conditions can negatively impact later developing fundamental motor skills, adaptive functioning, language, and social communication (4–7).

Clinically, evidence suggests that atypical movement and coordination could be an important factor in the early diagnosis of neurodevelopmental conditions (8). The onset of walking is one major developmental milestone that allows children to travel farther, faster, and engage with their larger environment (9). Studies have shown that children with autism may show delayed walking onset or atypical features of walking regardless of when the milestone is achieved (10, 11). Delays and atypical walking can impact the child's overall developmental functioning and limit opportunities for participating in activities and engaging with peers (12). Given the prevalence and pervasiveness of motor impairments in NDDs, there is a need to improve the identification, treatment, and surveillance of these motor impairments early in life and over time. Furthermore, individuals with NDDs have heterogenous presentations with a range of intellectual and verbal ability and co-occurring behavioral diagnoses. Commonly used standardized assessments of motor function in these populations often have limitations due to the level of cognitive ability or attention needed to understand and complete the tasks (13, 14). The use of quantitative tools such as motion tracking, wearable sensors, or pressure sensor gait analysis methods, can overcome these limitations as they alleviate this cognitive burden and provide objective and granular measures of movement abilities (15). There are also considerations of using methods that can be employed in the home environment because these methods can capture naturalistic movements and be more easily used longitudinally to capture change over time or secondary to an intervention.

In recent years, markerless computer vision technologies have shown rising promise for use in motor analysis. “Markerless” refers to technologies that do not rely on wearable reflective markers, unlike optical motion capture (3D motion capture). A growing body of research has tested video-based gait analysis using two-dimensional pose estimation to automatically track anatomical landmarks such as knees or ankles in digital videos. Video-based gait analysis has been evaluated against established methodologies such as motion capture and several studies have reported accuracy within established thresholds (16). In particular, OpenPose has emerged as a free, popular open-source software that can be used by researchers to perform pose estimation (17). Previous research has tested the validity of pose estimation for gait analysis by recording adults walking on a treadmill while concurrently measuring their gait with a marker-based motion capture system. In this case, researchers found that knee and joint angles calculated with OpenPose were significantly correlated with those calculated with motion capture (18). Another study compared OpenPose analysis of adult gait with simultaneously recorded 3-D motion capture and found that spatiotemporal gait parameters were accurate and could be used to measure change (19).

Recent research has expanded on the body of evidence validating pose estimation by testing its real-world applications in clinical and non-clinical settings. This includes clinical motor assessments for patients with degenerative or developmental conditions such as Parkinson's Disease and cerebral palsy, as well as limited use in infant locomotion (20). For example, spatiotemporal gait parameters of stroke patients walking on a treadmill and overground calculated with pose estimation are significantly correlated with motion capture (21). The successful implementation of video-based pose estimation presents a promising approach to mitigate many of the barriers associated with common motor analysis methods. Only a smartphone or tablet is needed to record digital videos which significantly lowers cost compared to 3D motion capture systems. Furthermore, pose estimation enables at-home motor assessments using any type of handheld device (e.g., smartphone, tablet). These benefits serve to improve the overall scalability and reach of motor research, particularly for individuals with NDDs who may have geographic, physical, or behavioral barriers to accessing a research environment.

Despite the numerous applications and benefits of pose estimation that have been identified for motor analysis in adults, studies testing the feasibility and validity of this technology in pediatric populations, particularly children with NDDs, are extremely limited. Markerless methods are of particular interest for capturing data in young children who may be averse to wearing physical markers, a problem which is likely exacerbated by the presence of NDDs. Researchers have explored leveraging 2D pose estimation in machine learning models to predict autism diagnosis in toddlers based on behavioral traits classified in clinical screening tools, which has achieved an accuracy of up to 80% (22). Preliminary findings have also shown that a deep learning model can be trained to calculate spatiotemporal gait parameters from keypoints overlayed onto videos of children with cerebral palsy using pose estimation. Researchers found high correlation with motion capture for walking speed, cadence, gait deviation index, and knee flexion (23). These findings suggest that pose estimation could prove to be a valuable tool for developmental research, a field in which rigorous motor measures are essential for determining developmental milestones and identifying motor differences in clinical populations who have atypical development.

Considering the totality of the current findings in the field, there are three major motivations for the present study:

1. Video-based gait analysis with pose estimation can be captured in any context compared to laboratory based measures such as pressure sensors or 3D motion capture with wearable markers, making it an attractive option for cost effectiveness and scalability.
2. Markerless methods are ideal for pediatric populations with NDDs, who may struggle to comply with wearing physical markers or sensors.
3. There is currently a scarcity of studies assessing the validity and generalizability of video-based gait analysis for young children with varying developmental diagnoses.

To address this gap, we leveraged OpenPose to perform pose estimation and conduct a retroactive video analysis of children during overground walking in which footfalls were simultaneously recorded by a ProtoKinetics Zeno walkway system, an instrumented gait mat. Pressure-sensing walkways are a gold standard measure for deriving spatiotemporal gait parameters and can serve as a reference when evaluating novel technologies (24). A custom workflow that has previously been validated in adult populations was used to calculate spatiotemporal gait parameters from videos that had been analyzed in OpenPose. This software is equipped with a user interface that allows researchers with limited training to review videos and correct for any errors in pose estimation prior to variable calculation (19). Spatiotemporal gait parameters were additionally calculated with the ProtoKinetics Movement Analysis Software (PKMAS), the Zeno walkway's associated gait analysis software. Our primary goals are to:

1. demonstrate the feasibility of using pose estimation to perform gait analysis in children with and without neurodevelopmental conditions; and
2. assess the validity of OpenPose for measuring spatiotemporal gait parameters through comparison to PKMAS.

Specifically, we focus on toddlers with a diagnosis of autism, non-autism developmental concerns, a genetic neurodevelopmental syndrome, or typical development. We hypothesized that OpenPose analysis would yield spatiotemporal gait parameters that were significantly correlated with those calculated using the instrumented gait mat.

## Methods

### Ethics statement

Our study protocol and data collection methodology were approved by the University of California, Los Angeles (UCLA) Institutional Review Board (IRB#17-001269, IRB#17-001265, IRB#17-000262). Due to the age of the participant population and/or diagnoses that affect cognitive abilities, a legally authorized representative of all participants provided written informed consent for their data to be used in related research.

### Participants

Participants were recruited from ongoing longitudinal research studies at the UCLA Center for Autism Research and Treatment and included children with autism, non-autism developmental concerns, typical development, tuberous sclerosis complex (TSC), or 22q deletion. Individuals with autism were diagnosed using validated assessments including the autism diagnostic observation schedule, second edition (25) and the autism diagnostic interview-revised (26). Individuals with TSC were diagnosed either through genetic testing or meeting clinical criteria for TSC and the individual with 22q deletion was diagnosed through genetic testing. All participants completed the same standardized gait data collection protocol between April 2018 and July 2023.

### Developmental assessment

Developmental level of participants was evaluated using the Mullen Scales of Early Learning (27). The Mullen Scales of Early Learning is a widely used developmental assessment that provides information on several subscales, including (1) fine motor, (2) visual reception, (3) receptive language, (4) expressive language, and (5) gross motor. The first four of these subscales can be used to compute an early learning composite score, which is a measure of overall intelligence. The Mullen was administered by trained clinical psychologists. We excluded Mullen data that was collected more than 3 months away from the participant's gait assessment.

### Data collection using pressurized gait mat and video recordings

Spatiotemporal gait parameters were captured using the ProtoKinetics Zeno Walkway, a 16 ft mat with embedded pressure sensors sampling at a rate of 120 Hz, and analyzed with PKMAS. Participants walked four full lengths of the mat plus an additional four feet on either end to account for acceleration and deceleration. Participants walked using their spontaneous self-paced gait. Due to the young age of the participants, a researcher gave a demonstration and further instruction when necessary to elicit this type of walking. Additional passes were performed to replace passes where the participant stepped off the mat, did not use a self-paced gait (e.g., running, jumping, scooting, shuffling), or stopped on the mat in the middle of a pass. If a researcher determined that sufficient attempts had been made but the child did not complete four passes using a self-paced gait, the trial was concluded and analyzed so long as at least two passes could be used to generate average spatiotemporal gait parameters.

**Figure 1:**
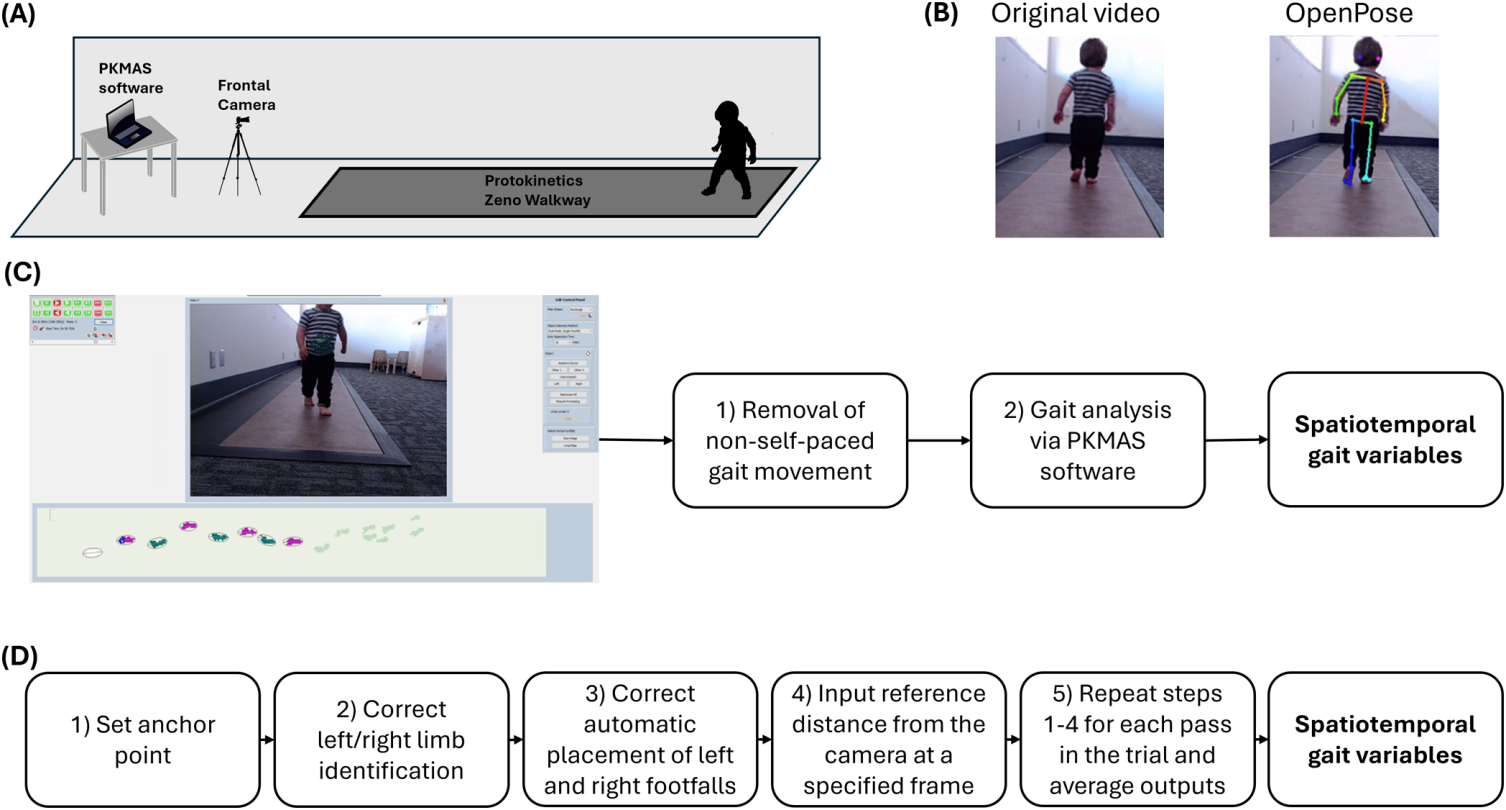
Experimental design. Includes a general overview of the experimental design used for gait data collection, including **(A)** the experimental setup consisting of a 16-foot instrumented gait mat, a frontal plane camera, and gait analysis software; **(B)** examples of a video before and after OpenPose analysis; **(C)** the steps for calculating spatiotemporal gait variables using the PKMAS software; and **(D)** the pipeline for calculating spatiotemporal gait variables using pose estimation.

Trials were simultaneously recorded with a monocular camera that was centered and placed five feet away from the edge of the mat, parallel with the walking direction of the participant (i.e., the videos provided a frontal view of the participants walking toward or away from the camera). A visual representation of the experimental setup can be seen in Figure 1A. Trials were processed, reviewed, and cleaned within PKMAS by trained research assistants to ensure that the recorded data consisted only of the participant's spontaneous self-paced gait. PKMAS automatically detects right and left footfalls and displays them on a graphical representation of activated sensors, which is synced with the recorded video. Spatiotemporal gait parameters were derived through the following steps: (1) review the video for movement that did not match the participant's spontaneous self-paced gait, such as running, jumping, crawling, or stopping, and manually mark the corresponding sensor activations for exclusion, and (2) run the PKMAS software to generate mean spatiotemporal gait parameters for each trial (Figure 1C).

### Video review

Review criteria were developed to verify that retrospectively recorded gait videos were compatible with video-based gait analysis. In PKMAS, videos were used for manual review when cleaning data and the angle, tilt, or distance of the camera relative to the gait mat did not affect variable calculation. However, these are important factors for video-based pose estimation. The purpose of developing review criteria was to ensure consistency between videos recorded at different time points so that spatiotemporal gait parameters could be accurately calculated. Criteria included camera angle, camera frame, camera distance, and pass quality (further detail on each provided below). When discussing these criteria, we use “footfall” when referring to the contact of a single foot with the mat, “pass” when referring to a group of consecutive footfalls moving in a single direction (toward or away from the camera), and “trial” when referring to the full collection of passes in a video recording. At least two passes fulfilling all four criteria were needed to calculate average spatiotemporal gait parameters for a trial. Any passes that did not meet one or more of these criteria were not analyzed or included in variable calculation.

#### Camera angle

To fulfill the camera angle criterion, the camera must have been placed parallel with the mat to show a centered, frontal view of the participant. This placement was necessary because our software used to calculate spatiotemporal gait parameters is optimized for purely frontal and/or sagittal views (19). Thus, a video in which the camera is angled in other directions may not yield accurate variables.

#### Camera frame

The camera frame criterion required a non-obstructed view of the participant's full torso and lower limbs. For example, a person entering the field of view between the camera and the participant would cause an occlusion and preclude accurate tracking of the participant. Furthermore, the pixel height of the participant's torso was a necessary variable for generating spatiotemporal gait parameters, which was the motivation for requiring a view of the participant's full torso and lower limbs. Passes containing brief camera obstructions could still be used so long as at least 5 consecutive steps had an unobstructed view of the participant.

#### Camera distance

To meet the camera distance criterion, we had to identify a frame in the video with a known distance from the camera to the participant, which is a necessary variable for calculating spatiotemporal gait parameters from frontal videos. This was possible because the gait mat and camera were set up in a standardized and consistent manner per protocol, with a known measured distance from the camera to the start and end of the gait mat. Thus, a video met the camera distance criterion if it contained a frame where the participant was at the beginning or end of the mat.

#### Pass quality

The pass quality criterion was created to ensure comparability between spatiotemporal gait parameters generated using PKMAS and those derived from pose estimation outputs. In PKMAS, data were cleaned by reviewing the video and manually marking activated sensors for exclusion if they were not part of the participant's spontaneous self-paced gait. A pass met the pass quality criterion if it contained five or more consecutive steps that had not been excluded during data cleaning. Any trials with a corrupted or missing video file did not satisfy the pass quality criterion by default. To guarantee that no discarded gait data was included in videos used for pose estimation, each pass was exported from PKMAS as a separate video containing only the first to last foot contact.

### Video-based gait analysis

#### Openpose analysis

As a preliminary step, the video files exported from PKMAS were converted from .wmv to .avi using Shutter Encoder, a free video conversion software. This step was necessary to ensure compatibility with both OpenPose (the freely available human pose estimation algorithm used in this study), and the custom software used to calculate spatiotemporal gait parameters. Shutter Encoder was also used to adjust the contrast and brightness of some videos that had poor lighting, which helped improve the quality and accuracy of pose estimation.

**Figure 2:**
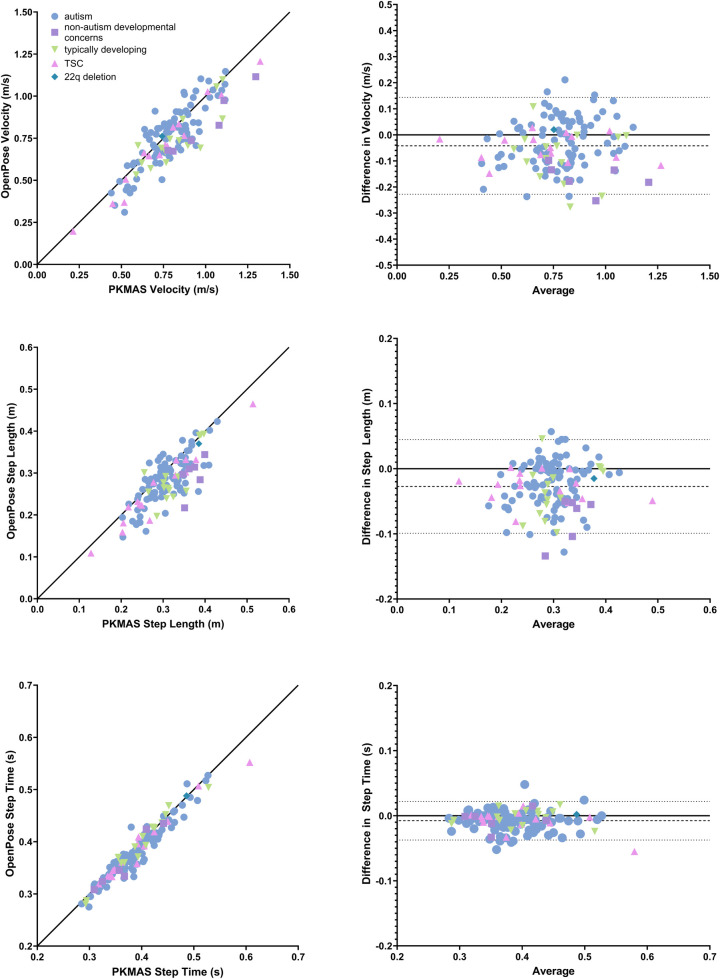
Comparison of spatiotemporal gait parameters between OpenPose and PKMAS. Displays Pearson correlations and bland altman plots (difference vs. average) for velocity, step length, and step time. Pearson correlations include a line of identity and Bland Altman plots include average difference and 95% confidence intervals, represented by dashed and dotted lines, respectively. All plots are grouped by participant diagnosis.

Frontal plane gait videos were then processed in Python software running OpenPose analysis locally through Anaconda Navigator. We employed the BODY_25 model to overlay and track 25 keypoints corresponding with various anatomical landmarks onto persons detected within the video frame (17). These include nose, neck, eyes, midpoint between eyes, ears, shoulders, elbows, wrists, hips, knees, ankles, heels, big toes, and small toes. The resulting OpenPose analysis yielded (1) a video file displaying a visual representation of the keypoints overlaid upon the original video as shown in Figure 1B and (2) JSON files containing the time series data for pixel coordinates associated with each keypoint in the video.

#### Analysis of spatiotemporal gait parameters

Spatiotemporal gait parameters were calculated using a custom MATLAB codebase created by researchers to generate spatiotemporal gait parameters from OpenPose keypoints (28). Leveraging this existing pipeline allowed for fast, intuitive calculation of variables that have been reliably captured in other populations. Upon selecting the appropriate video and JSON files, the following steps were followed to generate gait parameters: (1) select an anchor point to choose the individual that should be tracked when multiple persons are present, (2) correct identification of right and left shoulders and ankles that had been mis-identified by OpenPose, (3) inspect the automatic placement of right and left footfalls on a sinusoidal graph and make corrections when necessary, (4) select a reference frame and input the distance from the camera to the participant at that frame, and (5) repeat steps 1–4 for each pass in the trial and average the outputs (Figure 1D).

### Statistical analysis

We compared PKMAS and OpenPose variables using the absolute difference of the means for velocity, step length, and step time. In this study, velocity is defined as the speed of the participant in meters per second and step time is the time between footfalls in seconds. Step length was determined slightly differently between PKMAS and OpenPose, with the former measuring the distance between the center of two footfalls in centimeters and the latter relying on the distance travelled by the torso between footfalls. Spatiotemporal gait parameters were compared between PKMAS and OpenPose through Pearson correlations. Two-tailed *p* values were computed with 95% confidence intervals. A Bland-Altman analysis was used to assess the agreement between OpenPose and PKMAS by comparing the difference (OpenPose – PKMAS) to the average of each data point (29). The absolute difference of means was reported for velocity, step length, and step time. As videos were captured with a frequency of 30 Hz, all variables were reported with a precision of one centimeter or ten milliseconds.

## Results

Participants completed four passes on an instrumented gait mat with simultaneous video recordings of the frontal plane, where one pass constitutes walking from one end of the mat to the other. 128 videos were analyzed out of 192 total videos. Common reasons for excluding videos were the camera not being positioned parallel with the gait mat, low participant adherence to the data collection protocol, and missing or corrupt video files (see methods for full details on exclusion criteria).

Our analysis included 112 unique participants who had autism (*n* = 77), typical development (*n* = 15), TSC (*n* = 13), non-autism developmental concerns (*n* = 6), or 22q deletion (*n* = 1). The average age across all time points was 30 months, and all children were between the ages of 14 and 65 months (Table 1). Developmental data from the Mullen Scales of Early Learning was included for 99 participants who had testing administered on the same day as the gait assessment (*n* = 91) or within 3 months of the gait assessment (*n* = 8). Data was excluded for 13 participants who either did not complete the Mullen (*n* = 3) or had the Mullen administered greater than 3 months from the date of their gait assessment (*n* = 10). The gross motor subscale was not administered to participants older than 36 months and is thus excluded from our analysis due to missing data. A summary of the visual reception, fine motor, expressive language, receptive language, and early learning composite scores can be seen in Table 2.

**Table 1:** Participant demographics.

Group	Participants			Trials	Age (months)	Height (cm)	Weight (lb)
	*n* (%)	Male (%)	female (%)	n (%)	Mean (sd)	Mean (sd)	Mean (sd)
Total	112 (100.0%)	74 (66.1%)	38 (33.9%)	128 (100.0%)	30 (8)	92.41 (7.28)	30.53 (5.22)
Autism	77 (68.8%)	57 (50.9%)	20 (17.9%)	91 (71.1%)	29 (7)	92.48 (6.50)	30.42 (4.76)
Typically developing	15 (13.4%)	7 (6.3%)	8 (7.1%)	15 (11.7%)	33 (11)	92.83 (8.82)	31.34 (6.11)
TSC	13 (11.6%)	5 (4.5%)	8 (7.1%)	15 (11.7%)	28 (13)	90.20 (10.48)	29.51 (6.98)
Developmental concerns	6 (5.4%)	4 (3.6%)	2 (1.8%)	6 (4.7%)	31 (6)	94.83 (5.35)	31.60 (5.32)
22q deletion	1 (0.9%)	1 (0.9%)	0 (0.00%)	1 (0.8%)	43 (0)	99.00 (0)	37.40 (0)

Displays the number of unique participants, trials, and average age in months. As some participants were assessed at multiple timepoints, means and standard deviations for age, height, and weight have been calculated based on the number of total trials.

**Table 2:** Overview of participants’ developmental level.

Group		Visual reception	Receptive language	Expressive language	Fine motor	Early learning composite
	*n*	Mean (sd)	Mean (sd)	Mean (sd)	Mean (sd)	Mean (sd)
Autism	72	39.56 (16.98)	34.26 (16.43)	35.25 (16.17)	37.15 (14.36)	76.46 (25.79)
Typically developing	13	59.62 (7.332)	56.54 (9.171)	54.31 (10.09)	49.23 (9.82)	109.7 (13.40)
TSC	8	26.38 (9.023)	25.75 (7.778)	26.00 (6.188)	29.38 (10.78)	59.13 (10.37)
Developmental concerns	6	52.33 (16.32)	52.33 (7.891)	45.33 (12.14)	51.50 (16.26)	101.0 (15.49)

Overview of average developmental level for each diagnostic group, as determined by the Mullen Scales of Early Learning. The 22q deletion category is excluded from this table due to not having Mullen data available. Means and standard deviations for T-scores are reported.

The absolute difference of means between OpenPose and PKMAS was 0.04 m/s (sd = 0.10 m/s) for velocity, with 95% limits of agreement ranging from −0.23 to 0.14 m/s. The absolute difference of means for step length was 0.03 m (sd = 0.04 m) with 95% limits of agreement ranging from −0.10 to 0.05 m. For step time, the absolute difference of means was 0.01 s (sd = 0.02 s) with 95% limits of agreement ranging from −0.04 to 0.02 s (Figure 2). Statistically significant positive correlations were found between OpenPose and PKMAS for velocity (*r* = 0.87, *p* < 0.0001), step length (*r* = 0.79, *p* < 0.0001), and step time (*r* = 0.96, *p* < 0.0001).

## Discussion

We found that markerless video-based gait analysis using pose estimation is feasible and produces valid gait data in children with and without NDDs. Additionally, we were able to demonstrate that meaningful gait data could be ascertained from children with a large range of developmental abilities. As we hypothesized, our results demonstrate a strong agreement between OpenPose and PKMAS for velocity, step length, and step time, which were characterized by statistically significant correlations. We now review in detail the comparison of gait parameters derived in PKMAS compared to OpenPose.

Step time showed the highest agreement with PKMAS when calculated using pose estimation, with an absolute mean difference of 0.01 s and a Pearson correlation coefficient of 0.96. Velocity and step length also showed high agreement with PKMAS, albeit to a lesser degree than step time. The absolute mean difference for velocity and step length were 0.04 m/s and 0.03 m, respectively, with Pearson correlation coefficients of 0.87 and 0.79. Both variables could have been affected by factors that influenced the accuracy of step length calculation using pose estimation. The codebase used to calculate spatiotemporal gait parameters after performing pose estimation measured step length as the distance travelled by the torso between consecutive bilateral heel strikes. Conversely, in PKMAS these variables are measured from the center of each footfall. Previous studies employing the same analysis methods also found that videos with a sagittal view of overground walking were more accurate for calculating step length compared to frontal-plane videos that were used in this study (19). It is also possible that there was greater variability in calculating step length resulting from both methodological and developmental considerations in the pediatric participant population. Due to body and foot size, children are represented by fewer pixels within the frame of a video. Consequently, small deviations in keypoint tracking can have a greater impact on step length calculation compared to adult populations (30). This interpretation is supported by previous findings that pose estimation is less accurate at determining step length when a participant is further from the camera, which is a similar effect to a participant being physically smaller in size (19). As velocity is a function of both step time and step length, it follows that any improvements to step length accuracy would in turn improve accuracy for velocity.

A major strength of the present study is the evaluation of gait in a sample of children between the ages of 1 and 5 years. From our review of the literature, very few studies comparing spatiotemporal gait variables obtained from a pose estimation model to an established methodology have included children younger than six and none have included children younger than three, which is a critical period in gait formation and development. Prior studies have demonstrated that different pose estimation models (KAPAO, Detectron2) are able to detect significant differences in gait characteristics based on age or diagnosis, with one preprint including children as young as three in their sample (31, 32). Another study compared gait characteristics generated with OpenPose to 3D motion capture data of typically developing children to predict Duchenne muscular dystrophy with up to 97% accuracy (33). We identified only three studies that directly compared OpenPose or a similar pose estimation model to an established methodology (3D motion capture) for gait analysis in children as young as three, all of which found significant correlations or non-significant differences between methods (23, 34, 35). Thus, our findings contribute to a growing body of work demonstrating the utility of video-based gait analysis using pose estimation in pediatric populations while providing the first evidence that valid results can be obtained from the initial onset of gait (Table 3).

**Table 3:** Related works.

Reference	Developmental group	Mean age (sd)	Sample size	Gait features analyzed	Methods used	Key findings
Present study	Autism, developmental concerns, TSC, 22q deletion, typically developing	2.5 (0.67)	128	Velocity, step length, step time	OpenPose vs. Instrumented gait mat	Significant correlations ranging from 0.79 for step length to 0.96 for step time
(23)	Cerebral palsy	11.00 (5.90)	1,792	Velocity, cadence, knee flexion, gait deviation index	OpenPose vs. 3D motion capture	Significant correlations ranging from 0.73 for velocity to 0.83 for knee flexion
(31)^a^	Typically developing	6.70 (2.70)	198	Extension, flexion, and range of motion for hip and knee, swinging angle	KAPAO	Detected significant differences in gait features between age groups
(32)	16p11.2 mutation, typically developing	9.90 (3.27) 9.67 (4.00)	15 12	Gait synchrony, balance	Detectron2	Detected significant differences in gait synchrony and balance between groups
(33)	Duchenne muscular dystrophy, typically developing	– (–) 9.38 (–)	55 21	Velocity, step length, stride time, cadence, flexion of hip and knee	OpenPose, 3D motion capture	97% prediction accuracy of Duchenne muscular dystrophy
(34)	Typically developing	13.00 (–)	20	Joint angles of hips, knees, and ankles	OpenPose vs. 3D motion capture	Differences were non-significant for the knees, but significant for hips and ankles
(35)	Developmental hip dysplasia, typically developing	6.08 (2.22) 6.22 (2.02)	10 18	Extension, flexion, and range of motion for hip and knee	KAPAO vs. 3D motion capture	No significant differences between methods

Comparison of findings in the present study with other studies applying markerless methods to gait analysis in children. Age and sample size are listed separately by developmental group in some cases as this was how it was reported in the corresponding study. Means or standard deviations listed as — were not reported.

^a^ The article is a pre-print.

When considering applications for pose estimation, it is useful to consider accuracy relative to minimum clinically important difference (MCID). However, it is important to note that reliable MCIDs for spatiotemporal gait parameters have not been clearly defined in the literature, especially in children. Previous studies have reported the MCID for velocity to be between 0.04 and 0.1 m/s in older adults, and 0.08 m/s in adults with Parkinson's (36–38). It is known that younger children have greater gait variability (39), which would suggest that a higher MCID for velocity is likely needed when assessing pediatric populations. In our sample, the mean difference for velocity was below 0.1 m/s in 65% of trials, with a sample mean of 0.04 m/s and limits of agreement ranging from −0.23 to 0.14. While these results are promising within the context of existing research on MCID for spatiotemporal gait parameters, further research is needed to clearly establish MCID thresholds in pediatric populations. Comparison of gait analysis methodologies in a larger sample may aid in accomplishing this goal.

There were some limitations in the present study which should be considered when interpreting the results. As previously mentioned, our study was a retrospective analysis of frontal gait videos. Thus, we were unable to test different camera angles and compare the accuracy of spatiotemporal gait parameters. Prospectively designed studies can optimize data collection for video-based pose estimation by simultaneously capturing frontal and sagittal videos. As this was a single-site study, we are also unable to speak to comparability of results across locations that may have varying recording conditions. Future studies with larger sample sizes conducted across several locations could further expand our understanding of the variability of pediatric gait and how measurement using pose estimation models compared to other methods may accurately account for this variability. Lastly, the present study was not designed to prescribe an optimal method of markerless video-based gait analysis in young children. Many pose estimation models are available (16), and further efforts should explore multiple methods and compare results to determine the protocol that yields highest accuracy.

Our results show that video-based gait analysis using pose estimation can provide reliable and valid gait data in toddlers with and without NDDs. This tool is promising for improving access to developmental research, as data collection can be completed with accessible video recording devices and minimal data collection methods (e.g., measurement of distance from camera to starting point, demonstration of walking trials). Additionally, these methods can be employed in more naturalistic environments such as a participant's home. It follows that video-based gait analysis presents fewer barriers compared to other gait assessment methodologies, as it is low cost, does not require participants to travel to a controlled clinic or lab setting, and does not use wearable markers that could pose challenges for some participants. Furthermore, our results demonstrate that the methods used in this study are feasible and accessible for children with a large range of developmental abilities to complete, which increases our understanding of gait in those with a range of developmental abilities. These advantages open possibilities for using repeated measures to increase our knowledge of how gait ability changes over time in pediatric populations and improve clinical screening tools, particularly in those with NDDs where motor impairments are highly prevalent.

## Data Availability

The raw data supporting the conclusions of this article will be made available by the authors, without undue reservation.
